# Increasing a hospital-based violence intervention program’s referrals for children and families in a pediatric emergency department

**DOI:** 10.1186/s40621-025-00578-w

**Published:** 2025-05-02

**Authors:** Narmeen I. Khan, Sri S. Chinta, Brooke M. Cheaton, Mark Nimmer, Michael N. Levas

**Affiliations:** 1 Department of Pediatrics (Emergency Medicine), Medical College of Wisconsin, Milwaukee, WI 53226 USA; 2 Children’s Wisconsin, Milwaukee, WI 53226 USA; 3 Department of Pediatrics (Emergency Medicine), Medical College of Wisconsin Children’s Corporate Center, Suite C550 999 N. 92nd St, P.O. Box 1997, Milwaukee, WI 53201-1997 USA

**Keywords:** Hospital-based violence intervention program, Emergency department, Quality improvement, Crime victim advocate, Electronic medical record

## Abstract

**Background:**

Our pediatric tertiary care hospital sees a high rate of firearm injuries. Hospital-based violence intervention programs (HVIPs) reduce violent injury recidivism rates in victims. However, significant gaps exist in the delivery of trauma-informed services to families. Our specific aim was to increase our HVIP referral rate by 20% over a 12-month time frame for children seen for interpersonal violence in the emergency department (ED).

**Methods:**

Our quality improvement study was done at a pediatric tertiary care hospital and encompassed patients 0 to 18 years of age who presented to our ED for assault-related concerns from December 26, 2021 to June 23, 2024. The primary outcome measure was percentage of HVIP-eligible patients who received a referral from the ED. We conducted a root cause analysis by interviewing stakeholders including HVIP staff, ED providers, nurses, and social workers to understand gaps in the referral process. Key drivers included electronic medical record (EMR) trigger tools for referral placement, accessibility of HVIP staff, and staff knowledge of HVIP eligibility and services. We integrated three main EMR-based interventions on June 15, 2023 that triggered referrals to the HVIP.

**Results:**

Our ED HVIP referral rate during the pre-intervention period (December 26, 2021 to June 15, 2023) was 53.6%. During our post-intervention phase (June 15, 2023 to June 23, 2024), the referral rate reached and sustained at 93.5%, a 74.4% increase.

**Conclusions:**

We identified a large percentage of missed HVIP-eligible referrals and developed interventions that significantly increased our referral rate. However, this did not translate into increased enrollment, indicating the need for additional efforts.

## Introduction

More than 11,000 children ages 1 to 17 years have been killed by firearm-related injuries across the United States (US) from 2019 to 2023, regardless of intent [1]. Since 2000, the US has seen a rise in pediatric firearm-related deaths [2]. Firearms have become the leading cause of death in children in the US, with the majority affecting adolescent Black males [3]. 

Our pediatric tertiary care hospital resides in Milwaukee County, Wisconsin which has one of the highest rates of firearm injuries in the US, with an average of 25.9 per 100,000 firearm-related deaths relative to 18 per 100,000 people in the US in 2023 [4, 5]. Similar to national trends, the majority of homicides seen in Milwaukee County and at our institution are a result of penetrating trauma and disproportionately affect Black male youth [6, 7]. Of the 986 traumas seen at our institution in 2023 (including penetrating and blunt traumas as well as thermal injuries), 781 (79.2%) were admitted to the hospital. Of those admitted, 125 (16.0%) were penetrating injuries, the majority of which were gunshot wounds. In that same year, our institution saw 149 patients who suffered from firearm injuries, the majority of whom were Black (117, 78.5%) and male (114, 76.5%).

In addition to increased mortality rates, both indirect and direct firearm violence exposure have led to negative impacts on mental health such as childhood anxiety and depression [8]. Past work has shown that being a victim of violence is a predictor of re-injury; in particular, being a Black male, coming from a disadvantaged socioeconomic background, and having mental health conditions are risk factors for violent traumatic re-injury [9]. 

There are only 63 hospital-based violence intervention programs (HVIPs) as of September 2024 within the Health Alliance for Violence Intervention across the US [10]. HVIPs have shown significant positive impacts on enrolled youth, with decreased likelihood of criminal involvement and improved healthcare cost effectiveness [11, 12]. These programs work to interrupt the cycle of violence through individual, family, and community-based prevention strategies including hospital response when a child seeks treatment following a violent injury.

Our hospital’s HVIP, Project Ujima, is a multidisciplinary collaborative support network that assists pediatric victims of violence and their families during and after hospitalization. When a child presents to the emergency department (ED), providers, nurses, or social workers can place a consult to Project Ujima. The program also works with trauma surgeons and other subspecialties when a child is admitted to that service. The program offers resources including mental health support, job security, and mentorship as these individuals attempt to recover from their trauma and navigate societal stressors. Past work has demonstrated reduced recidivism rates in those enrolled in Project Ujima (1%) relative to those not enrolled (8%) [13].

Despite the proven effectiveness of HVIPs in reducing injury recidivism, significant gaps have been identified in the delivery of trauma recovery services to eligible families presenting to our ED, such as lack of financial support, few needs assessments, and high participant attrition rates [14]. Recent efforts at our hospital noted these gaps via a needs assessment and developed interventions using quality improvement (QI) methodology such as educating providers, nurses, and social workers to increase referral rates to our HVIP [15]. Some of the gaps included lack of knowledge regarding Project Ujima’s services and nurses’ ability to place consults [15]. Team members developed interventions to increase awareness about the HVIP and ultimately developed a best practice advisory (BPA) alerted in triage to operationalize the process of nurses placing orders [15]. QI studies have shown that though education is critical to sustaining improvement, it is often insufficient [16]. 

Building on these past endeavors at our institution, our global aim was to increase HVIP services to patients and families who seek ED care for interpersonal violence. Our specific aim was to increase our HVIP’s referral rate by 20% over a 12-month time frame.

## Methods

This is a QI study at a large pediatric tertiary care hospital. We evaluated patients up to 18 years of age who presented to our ED with concerns for interpersonal violence such as penetrating trauma including firearm injuries and stabbings as well as motor vehicle collisions. Exclusionary criteria including those who are in police custody, involved with child protective services, or present to the ED with a sexual assault-related concern. Crime victim advocates (CVAs) receive a page whenever a consult order for Project Ujima gets placed by ED providers or nurses. Consults can also be placed by inpatient teams such as trauma surgery if a child is admitted. Outside of the hospital setting, referrals can also be placed by police and school professionals if needs are identified. During on-call hours, CVAs will arrive to the ED to have face-to-face contact with the patient and caregiver, explain the program, and encourage enrollment. When a page is received after-hours, the Project Ujima team reviews the case in the morning and either calls the family or, if a patient is admitted, meets the patient and family in the hospital.

Our QI work was exempt from institutional review board review.

We conducted a comprehensive root cause analysis to understand current state and perform a needs assessment to identify gaps in the referral process. To do this, we interviewed stakeholders and distributed surveys to ED nurses; providers (including attending physicians, resident physicians, and advanced practice providers); and social workers. To the residents and nurses, the open-ended questions asked about whether they know the existence and services of Project Ujima, whether they know how to place a consult order to the program, what barriers currently exist to placing that contact, and suggestions for improvement. One team member also shadowed CVAs during patient and caregiver interactions in the hospital and home settings to better understand the barriers encountered when working with families; this was done via a Gemba walk.

**Fig. 1:**
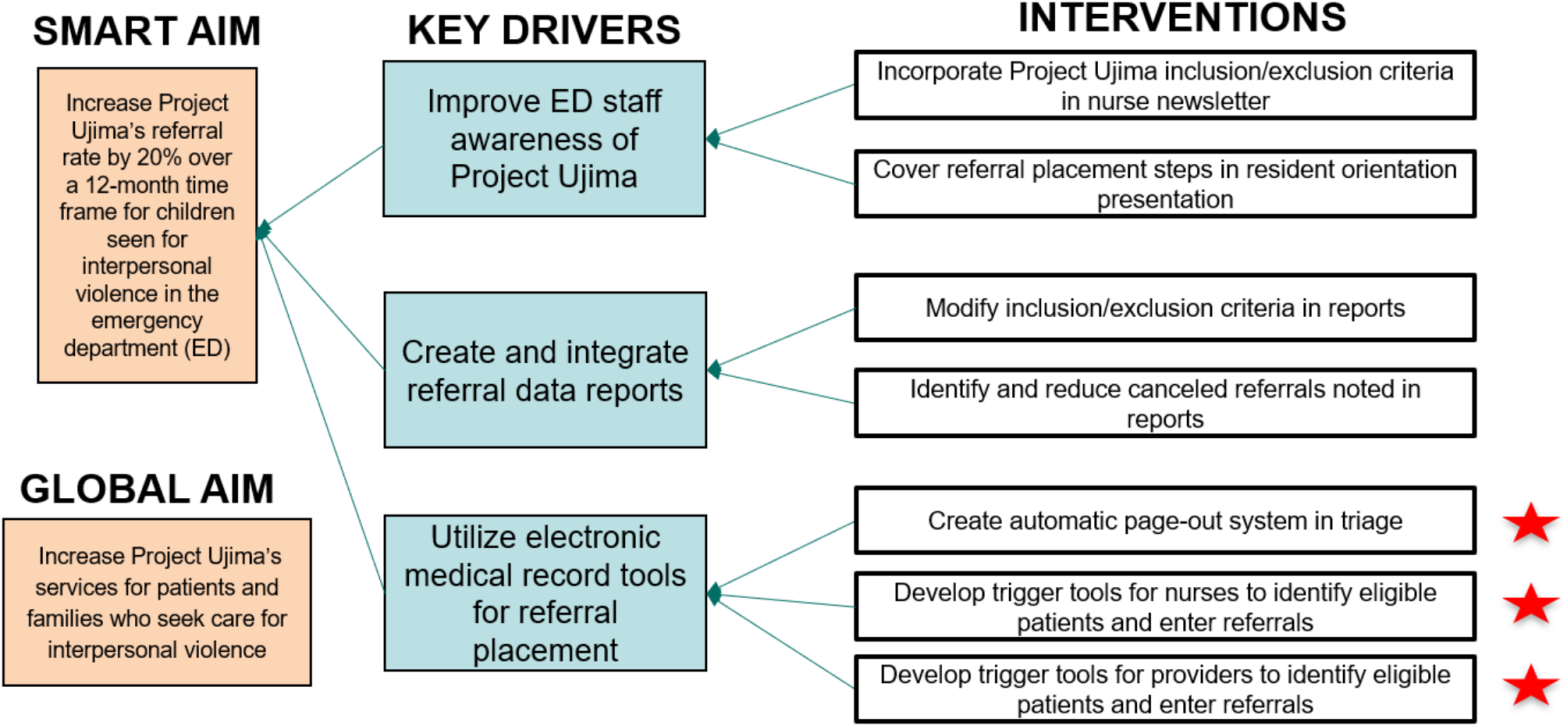
Key driver diagram with specific aim to increase Project Ujima’s emergency department referral rate. The red stars highlight electronic medical record interventions

Figure 1 shows our key driver diagram. Through our root cause analysis, we learned that key drivers included awareness from ED staff about the HVIP, integration of various reports that identified eligible patients, and electronic medical record (EMR)-based tools to trigger referrals. Our institution’s EMR is Epic. Interventions included teaching resident physicians about Project Ujima prior to the start of their ED rotation and working with our dedicated ED data analyst to integrate several referral data reports to obtain an accurate referral rate and identify missed referrals. Data was assessed at the visit level. We retrospectively reviewed de-identified patient data including chief complaint and ED disposition to create statistical process control charts and identify missed HVIP-eligible visits. We implemented one plan-do-study-act (PDSA) cycle to assess multiple interventions.

We focused a large portion of our efforts toward EMR tools that would trigger referrals, noted by the red stars in Fig. 1. A BPA was implemented in March 2021 which gets triggered in triage and leads to a Project Ujima consult order if the triage nurse answers “yes” to the following 2 questions when a patient checks in to the ED:

1. Is this visit a result of an injury?
2. (If answer to 1 is “yes”) Is this injury the result of an assault?

The BPA does not get triggered when a patient is in an active code or trauma, as this is distracting to the documenting nurse; once the patient is out of the resuscitation bay, the BPA can then pop up if criteria are met. If a patient leaves the trauma bay to go directly to the operating room or pediatric intensive care unit, the admitting service is expected to manually enter a consult to Project Ujima. This is often done in conjunction with social work consults.

Through our needs assessment, we learned from nurses that the BPA was disruptive to triage workflow, realizing that they have several other tasks that must be accomplished during triage and often do not have enough information about the patient to answer the above 2 questions. As an intervention, we worked with our EMR team to have the BPA alert the primary (instead of triage) nurse after a patient got roomed. This modification was well-received by nursing leadership and established June 15, 2023.

We also learned from our stakeholders the importance of provider input when placing HVIP referrals. Though any provider could enter a manual consult order in the past, we created a yellow banner that would be visible when an HVIP-eligible patient’s chart was opened which would remind providers to enter a consult order, similar to an influenza vaccine screening banner that was already established in our ED. This banner would only be visible if a nurse had not already entered a referral through the above BPA.

Furthermore, while learning more about the triage process, we discovered that our child life team receives automatic pages in triage for patients screened as having high anxiety. To mirror this, we worked with our EMR team to develop an automatic page-out system that would alert CVAs about HVIP-eligible patients in triage. This page occurred in addition to a consult order (which ultimately led to a page to the CVA), thus creating potential duplicate alerts with the BPA or yellow banner.

Our primary outcome measure was eligible referrals placed. Process measures included BPA-driven referrals, yellow banner-driven referrals, and automatic pages. Balancing measures included duplicate pages received by the CVA and number of ineligible referrals.

## Results

Some of the barriers identified by providers elicited in our surveys included not knowing what Project Ujima’s services are and whether a patient qualifies for those services. Furthermore, many providers mentioned relying on the social workers to place the referral.

Nurses also discussed how the BPA was less distracting. As discussed above, one suggestion included moving the BPA from triage to after a patient gets roomed so that the primary nurse caring for the patient, who knows more details about the patient and is not multi-tasking as robustly, can adequately respond to the BPA.

Social workers suggested having the referral process being in parallel, rather than in tandem, to social work consults. They brought up that often CVAs reach out to them without the social workers having done their assessment. Many also stated that the CVAs are more equipped in explaining Project Ujima services to patients and families than they are.

Via the Gemba walk, we learned the processes that take place when a patient arrives to the ED via triage or as a trauma. We discovered some of the barriers CVAs face when entering the ED and interacting with families, such as presence of time constraints (for instance, a child is about to be discharged home). These findings contributed to more buy-in to create the non-disruptive automatic paging system in triage.

**Fig. 2:**
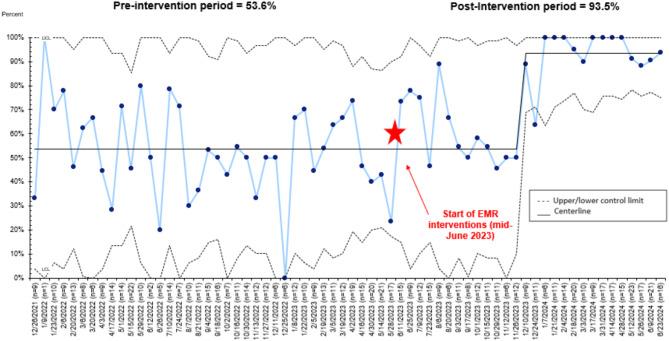
Percentage of visits with referrals to Project Ujima (p-chart). Numerator is all visits with a referral and denominator is all Project Ujima-eligible visits. Pre-intervention period is from December 26, 2021 to June 15, 2023. The red star represents the start of electronic medical record (EMR) interventions. Post-intervention period is from June 15, 2023 to June 23, 2024

Figure 2 is a p-chart showing percentage of visits for which a referral was placed from those eligible. In our pre-intervention period from December 26, 2021 to June 15, 2023, we had 53.6% referrals (305/569). In our post-intervention period from June 15, 2023 to June 23, 2024, our referral rate increased to 93.5% (202/216), a 74.4% increase.

Table 1 highlights our process measure data which shows the 3 modes through which a referral is placed (yellow banner-based referrals, automatic pages, and BPA-based referrals). In addition, we noted a rise in both balancing measures: there were ineligible referrals placed and more duplicate pages sent to the CVA for the same patient during the post-intervention period.

**Table 1:** Project Ujima referrals by mode

Year	Month	Yellow banner	Automatic pages	BPA
2023	June	0	0	0
2023	July	0	0	0
2023	August	0	0	0
2023	September	0	0	0
2023	October	0	0	0
2023	November	0	18	18
2023	December	0	22	22
2024	January	0	0	26
2024	February	0	0	36
2024	March	0	0	19
2024	April	2	0	37
2024	May	1	0	38
2024	June	2	0	28

Referrals to Project Ujima by mode in the post-intervention period (June 15, 2023 - June 23, 2024). BPA, best practice advisory

Table 2 shows a breakdown of firearm-related injuries to total referred to Project Ujima during the pre- and post-intervention periods. This data includes all types of referral venues, including ED and inpatient.

**Table 2:** Percentage of firearm and total referrals to Project Ujima

	Time frame	Firearm-related referrals	Total referrals	Percent firearm injuries of total referrals
**Pre-intervention period**	12/26/21–6/15/23	218	483	45.10%
**Post-intervention period**	6/15/23–6/23/24	108	415	26.00%

Firearm-related and total referrals to Project Ujima are shown for pre- and post-intervention periods. Referrals include all types of locations, including emergency department and inpatient

**Fig. 3:**
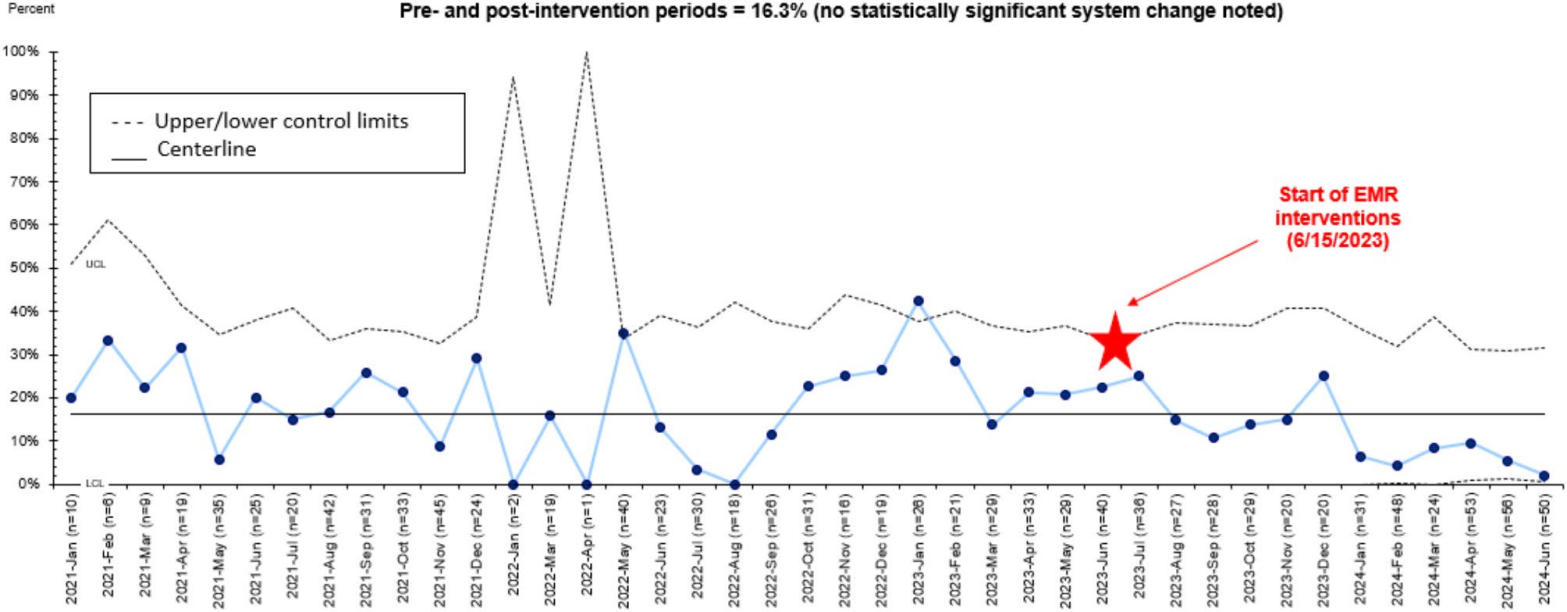
Percentage of patients enrolled into Project Ujima (p-chart). Numerator is all visits with an enrollment date and denominator is all appropriate Project Ujima referrals. Pre-intervention period is from December 26, 2021 to June 15, 2023. The red star represents the start of electronic medical record (EMR) interventions. Post-intervention period is from June 15, 2023 to June 23, 2024

Though we saw a significant increase in referrals after our interventions, our enrollment rate remained at 16.3% (183/1,123) during the pre- and post-intervention periods (17 and 12 months, respectively), showing no statistically significant system change, as we had not yet reached 8 points for the center line to shift by June 2024 (Fig. 3).

## Discussion

From our needs assessment, we learned that we are missing a large percentage of HVIP-eligible patients for referrals. We made modifications to our EMR that have led to sustained improvement in our HVIP’s ED referral rate. BPAs and other EMR tools have been utilized in past efforts to improve efficacy of healthcare delivery, including placement of referrals in other fields of medicine [17]. 

To date, few studies have shown efficacy of HVIP uptake. Enrollment rates rose from 2.4% when patients were admitted to 11.9% when approached in an outpatient setting [18]. Of 319 individuals eligible for an HVIP at a large trauma center between 2020 and 2022, only 12% were enrolled [19]. Both studies, however, did not speak to referral rates and whether this contributed to the low enrollment. Our study is unique in identifying barriers and bridging gaps to boost referral rates, ultimately allowing for increased enrollment into Project Ujima.

The results from our stakeholder surveys and Gemba walk were crucial to development of our EMR interventions. Gaining that insight, particularly from nurses that the BPA was distracting during triage, allowed us to optimize our interventions.

Extrapolating from Table 1 and after additional chart review, we discovered that CVAs were receiving most eligible pages using the automatic page-out system. One of our balancing measures was number of duplicate pages which usually occurred after the automatic page was sent out in triage and a referral was also placed via the BPA or yellow banner. To reduce this balancing measure, we worked with our EMR team to have the automatic page send a consult order and thus eliminate the BPA and yellow banner systems. This was achieved in July 2024 and has continued to sustain a high referral rate to date (over a 6-month period). The referral rate declined during a brief period after roll-out when we were still capturing BPA-based referral data into the denominator even though BPA no longer existed. With the automation process not being disruptive to workflow, we have also been able to incorporate visits with an active code or trauma, when previously the BPA did not trigger for those instances. We have received positive feedback regarding this intervention from all stakeholders, which we hope to apply to other ED-based injury prevention efforts. Future steps include performing more PDSA cycles to assess whether this recent intervention leads to a sustained increase in referral rate.

Firearm violence has always been a qualifying factor for referral to Project Ujima. While Milwaukee County has seen a decline in homicides, including firearm-related deaths since 2022 [6], Project Ujima has seen an increase in youth referred due to firearm exposure, regardless of intent. We are noticing an increase in the total referred as well in the post-intervention period. One reason may be that we are now capturing more patients through our EMR interventions who may not necessarily be eligible for Project Ujima (for instance, we are seeing patients who may suffered physical assault but are ineligible for Project Ujima). Thus, while we are seeing a rise in both numerator and denominator during recent years, the percentage of firearm-related concerns referred to Project Ujima has not significantly changed, as demonstrated by Table 2.

There are several limitations to our work. We rolled out all 3 EMR interventions at the same time (June 15, 2023) and did one PDSA cycle and thus were not able to realistically see the effects each had on the HVIP referral rate, despite having process control charts for each process measure. It would have been ideal to do multiple PDSA cycles with each intervention. Furthermore, though our efforts led to an increased referral rate, we realize that another important balancing measure is having enough Project Ujima staff to work with these eligible patients, whether it be in or outside of the hospital setting.

We also acknowledge that the pre-intervention period had large fluctuations in the referral process. We did not identify a particular cause for this, with the exception that perhaps referrals were not being consistently measured prior to the start of our interventions.

In August 2024, we worked with our EMR stakeholder to understand why automatic pages were no longer being captured from Epic into our statistical process control charts, showing 0 automatic pages from January 2024 to June 2024. We learned that the automatic pages were erased in production when the automatic page system became the primary means of Project Ujima consult placement. Though this issue was amended by our EMR stakeholder, we were not able to collect our previous data for automatic pages, as our control chart is updated using an Epic data pull on a biweekly basis.

Furthermore, we saw a decline in enrollment in our post-intervention period. This may be due to the denominator (number of eligible visits) increasing as we integrated and refined our data sets. One reason for a decrease in the denominator in the post-intervention period (Fig. 3) could be that our pre- and post-intervention periods vary in duration (17 and approximately 12 months, respectively).

We did not see a significant increase in enrollment (Fig. 3), thus not achieving our global aim. This may be due to the denominator (number of eligible visits) increasing as we integrated and refined our data sets. However, we far surpassed our specific aim of increasing our ED referral rate to Project Ujima by 20% by noting a rise of more than 70% after interventions.

Next steps include reaching out to patients and families to see why referrals get declined. We are working on a needs assessment and process map for what happens after a referral gets placed. Thus far, we have learned that referrals are often declined by families due to their vulnerable state in the ED soon after a triggering event such as a physical assault. We understand that learning about a program is not ideal after experiencing a traumatic event in the ED. Perhaps we can develop ways to share Project Ujima’s services to families when they are in a less vulnerable state.

## Conclusions

The interventions we developed using QI methodology led to a 74.4% increase in ED referrals to Project Ujima. However, this did not translate into increased enrollment, indicating the need for additional efforts.

## Data Availability

No datasets were generated or analysed during the current study.

## Abbreviations

US: United States
HVIP: Hospital-based violence intervention program
ED: Emergency department
QI: Quality improvement
BPA: Best practice advisory
CVA: Crime victim advocate
EMR: Electronic medical record
PDSA: Plan-do-study-act
